# Effect of Additives on Stability of Alumina—Waste Alumina Suspension for Slip Casting: Optimization Using Box-Behnken Design

**DOI:** 10.3390/ma12111738

**Published:** 2019-05-29

**Authors:** Milan Vukšić, Irena Žmak, Lidija Ćurković, Danko Ćorić

**Affiliations:** Department of Materials, Faculty of Mechanical Engineering and Naval Architecture, University of Zagreb, Ivana Lučića 5, HR-10000 Zagreb, Croatia; lidija.curkovic@fsb.hr (L.Ć.); danko.coric@fsb.hr (D.Ć.)

**Keywords:** alumina ceramics, waste alumina, slip casting, recycling, response surface methodology, optimization, additives, stabilization

## Abstract

The green machining of alumina (Al_2_O_3_) green bodies generates a certain amount of waste alumina powder. Waste alumina ceramic powder should be disposed of as non-hazardous waste in a legally compliant manner. The influence of additives on the stability of 70 wt.% (≈40 vol.%) alumina—waste alumina water-based suspension was investigated in the presented research. A Box-Behnken three-factor response surface design was used for the preparation of stable highly-concentrated suspensions with the addition of three additives. The optimal amount of each additive was selected according to the obtained results of minimal apparent viscosity: 0.05 wt.% Tiron as dispersant, 0.1 wt.% poly (vinyl alcohol) as binder and 0.2 wt.% magnesium aluminate spinel as abnormal grain growth inhibitor. The analysis of variance was used to identify the contribution of each additive. The zeta potential and sedimentations tests were performed to confirm the suspension stability measurements at different pH values. Alumina particles were optimally dispersed at pH values between 8 and 11. According to the results, the investigated composition of 20 wt.% waste alumina powder (weight content, dry alumina powder), with the addition of optimal amounts of additives, shows a possible application in the production of ceramics by slip casting.

## 1. Introduction

Alumina (Al_2_O_3_) ceramics constitute a very favorable material for different applications due to their very good thermal and chemical stability, high stiffness, high hardness, amazing wear resistance and low density. Other important properties include high electrical resistance combined with excellent heat conductivity. These properties allow the successful application of alumina products in various industrial, technical and military uses. Also, the excellent biocompatibility of alumina has enabled its increased potential for application in many medical applications, for example in dentistry, orthopedics, cardiology and bionics [1].

The production of alumina ceramics requires raw alumina powder. This powder is commonly produced from bauxite ore using the Bayer process. The environmental concerns of the rising amount of waste generated by the Bayer process have been considered as an important issue recently, despite the fact that this waste is considered non-hazardous [2]. Although the vast majority of the produced raw alumina is used for the production of aluminum metal, the same environmental concerns about producing raw alumina powder apply to the production of alumina ceramic products too.

Alumina ceramic products are commonly made by colloidal shaping methods. The green ceramic body achieved after drying is quite soft and easily machinable. On the other hand, the sintered ceramics are very hard and difficult to machine. Therefore, most, if not all, of the machining work on the ceramic part has to be done prior to sintering.

The process of ceramic green body machining generates waste ceramic powder which has to be disposed of. As per the European List of Waste (LoW) from 2000, this waste, coded as “10 12 03”, was described as “particulates and dust as wastes from the manufacture of ceramic goods, bricks, tiles and construction products” and considered as non-hazardous waste [3]. The European Commission Notice on Technical Guidance on the Classification of Waste from 2018 further labeled this waste as absolute non-hazardous (ANH) waste [4]. If not reused or recycled, non-hazardous waste may be landfilled at landfill sites specially prepared to protect the soil, groundwater and surface water from pollution by any leachates. Such landfills typically have a geological barrier and a bottom liner during the landfill use. The same barriers are applied on top of the landfill when the landfill is finally closed.

Besides the fact that the producers of alumina ceramic products have to pay fees for the disposal of alumina waste powder, this waste would better be reused in the production of novel products if possible. The European Waste Framework Directive from 2008 has defined the waste management hierarchy, i.e., the so-called 3R—“reduce, reuse, recycle”. This means that the first goal when dealing with the waste problem should be to prevent the waste from being created. Then, if the waste is created, it should be reused if possible for some other useful purpose. If reuse is not possible, the waste should be recycled, which means materially converted into some other useful material. Next, the non-recyclable waste is to be used for recovery, i.e., gaining energy. The disposal on legal landfills is advised as the very last option [5]. Similar actions and principles are encompassed in a more novel EU Circular Economy Action Plan from 2015, which proposes actions that should help to “close the loop” of a product lifecycle by reducing the generated waste, reusing the waste, and recycling where possible [6].

The presented research questions the possibility of use of waste alumina powder generated by green marching for shaping new ceramic products. The proposed shaping method was the slip casting technique.

Ceramic colloidal shaping methods are considered acceptable methods for achieving high-quality ceramic green bodies [7,8,9]. These methods include such well-known processes as slip casting and gel casting. Further, some novel processes, such as direct coagulation casting (DCC), have been suggested as methods to produce high-quality ceramic green bodies.

The colloidal suspension properties are influenced by the amount of solid phase content, the particle shape, as well as particle size distribution, as the most obvious factors. The other important parameter for colloidal suspensions are the interaction forces that occur among solid particles [10].

An understanding of the particle’s interaction mechanism is needed for the process optimization and for a better planning of the starting compositions. The first step in studying such mechanisms would be to achieve homogenously dispersed suspensions. The suspensions which have a high amount of solid content loading should have low the apparent viscosity in order to fill the mold more easily. Concentrated suspensions are monitored through their rheological properties, since these properties are the most important for the control of the shape forming process. In such a way, the rheological properties of concentrated suspensions determine the properties of the green bodies [11,12].

Slips are suspensions of ceramic particles in a liquid (usually water), containing varying amounts of dispersant, binder, sintering additives, etc. The slip casting method is a simple technique for the production of ceramic green bodies, which have high homogeneity and high density, and which may be applied to rather complex product shapes [13]. The production of ceramics by the slip casting method implies a technique in which the ceramic powder is mixed with deionized water and suitable additives. Ceramic particles generally do not mix well with water and tend to sediment and agglomerate. For this reason, it is necessary to use auxiliary substances, the so-called dispersants. Sometimes other sintering additives, such as binders and surfactants are added too. Finally, the green body is obtained by slip casting the prepared suspension into a mold. Gypsum is a good material for mold making since it is porous and quickly absorbs water [10,14].

The stability of a colloidal suspension influences the properties of the green body, which affect the properties of the sintered ceramic material. Modifying the ceramic powder surface properties and choosing the suitable dispersant, as well as optimizing its content, offer a possibility to reduce the apparent viscosity and hence avoid additional processing costs [15,16].

Among many commercially available dispersants, low-molecular-weight organic dispersants, such as disodium 4,5-dihydroxybenzene-1,3-disulfonate may be used when mixing alumina powder into water based solutions [17,18,19]. This compound, with the molecular formula of C_6_H_4_Na_2_O_8_S_2_, is also known by its commercial name Tiron. One of the studies has suggested the underlying principle for the suspension stabilization via Tiron. The organic molecules of Tiron adsorb on solid alumina powder, which creates a negative load on the ceramic particle surface. If the dispersant amount is beyond the optimum content, it will lead to the agglomeration of polymeric layers of the organic dispersant. The dispersant agglomeration will weaken the particle repulsion forces, and the apparent viscosity of the suspension will be enhanced [20].

The aim of the presented research was to investigate if waste alumina powder mixed with virgin alumina powder might be used for slip casting new ceramic products. The Box–Behnken response surface design was applied for the preparation of stable highly-concentrated alumina—waste alumina water based suspension by the addition of three additives: Tiron as the dispersant, poly (vinyl alcohol) (PVA) as the binder and magnesium aluminate spinel (MgAl_2_O_4_) for the prevention of abnormal grain growth. The experimental results were analyzed in order to find the optimal composition of water-based alumina suspensions with added waste alumina powder. Analysis of variance (ANOVA) was used to identify the contribution of individual additives. The stability of the suspension with the defined optimal composition was investigated by conducting rheological and zeta potential measurements and by sedimentations tests.

## 2. Materials and Methods

### 2.1. Preliminary Tests

**Figure A1:**
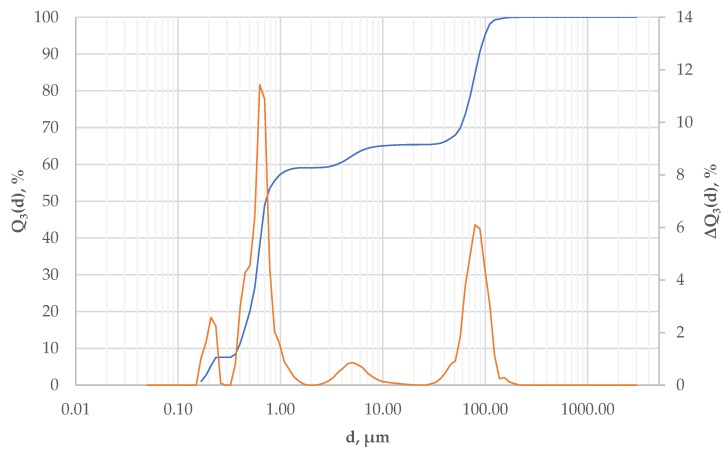
Particle size distribution for waste alumina powder.

Waste alumina powder obtained after green machining, with the average particle size 2.32–4.37 µm, and high-purity alumina powder, with the average particle size 300–400 nm (Alcan Chemicals, Stamford, CT, USA), were used in the presented study. The particle size distribution of waste alumina powder was determined in wet measurement mode by applying laser diffraction method (SALD-3101, Shimadzu, Kyoto, Japan). Before the measurement, waste alumina powder was pre-dispersed in deionized water. Figure A1 shows both the cumulative curve and the particle size distribution of the waste alumina powder expressed on the volume basis. The results indicate that waste alumina particles are in the size range from 0.2 to 214 µm.

Preliminary analyses of mixtures of Al_2_O_3_—waste Al_2_O_3_ with three different compositions were prepared in order to determine the optimal amount of waste alumina powder (15%, 20% and 25%). The commercial dispersant Tiron manufactured by Sigma-Aldrich Chemie GmbH, Steinheim, Germany was used to stabilize the highly concentrated alumina suspensions (Table A1).

### 2.2. Box-Behnken Response Surface Design

High-purity alumina powder with the average particle size 300 to 400 nm (Alcan Chemicals, Stamford, CT, USA) and waste alumina powder obtained by green machining were used. Mixtures of Al_2_O_3_—waste Al_2_O_3_ with an amount of 20 wt.% (weight content, dry powder) of waste alumina were prepared, as the preliminary results indicated (see Section 3.1. Preliminary Results). Based on the preliminary results, the addition of 20 wt.% of waste alumina has been chosen as a compromise between keeping the apparent viscosity relatively low and increasing the amount of reused waste alumina powder. Tiron from Sigma-Aldrich Chemie GmbH, Steinheim, Germany was used again for the stabilization of highly-concentrated water based suspensions of alumina, as suggested in literature [20]. The binder poly (vinyl alcohol) (PVA) manufactured by Sigma-Aldrich Chemie GmbH, Steinheim, Germany was added to the ceramic suspension in order to improve the strength of the green bodies, as proposed in other recently published studies [21,22]. Finally, magnesium aluminate spinel (MgAl_2_O_4_) made by Alfa Aesar, Haverhill, MA, USA was used to inhibit the abnormal alumina grain growth during the sintering process, as shown in [23].

A design of experiments (DOE) model has been developed in order to detect possible interactions between variables. Design-Expert 11.1.2 software of Stat-Ease, Inc. (Minneapolis, MN, USA) was used to obtain the Box-Behnken response surface design.

Water-based alumina suspensions with 70 wt.% (≈40 vol.%) of solid loading (mixture of alumina and waste alumina powder) were prepared by using different amounts of three additives:

1. 0.05 dwb.% to 0.15 dwb.% (“Dry Weight Basis”, i.e., weight content of dry powders) dispersant Tiron;
2. 0.1 dwb.% to 0.5 dwb.% poly (vinyl alcohol) (PVA) as binder;
3. 0.2 dwb.% to 1 dwb.% magnesium aluminate spinel (MgAl_2_O_4_) for the prevention the abnormal grain growth.

**Figure A2:**
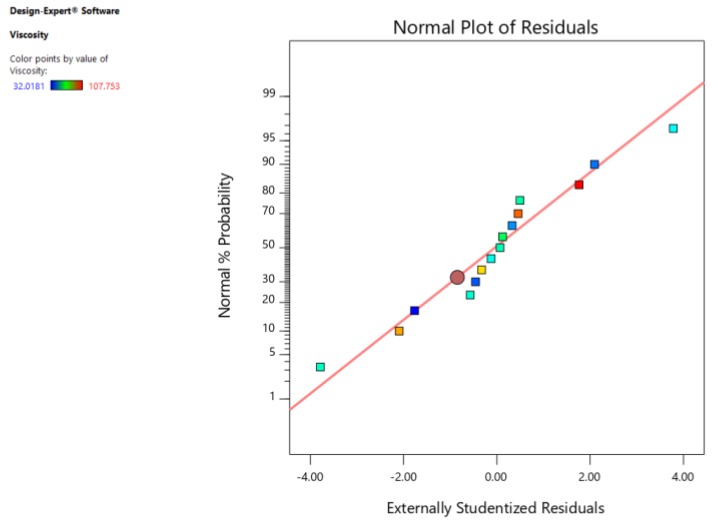
The normal probability plot of residuals versus predicted response values.

**Figure A3:**
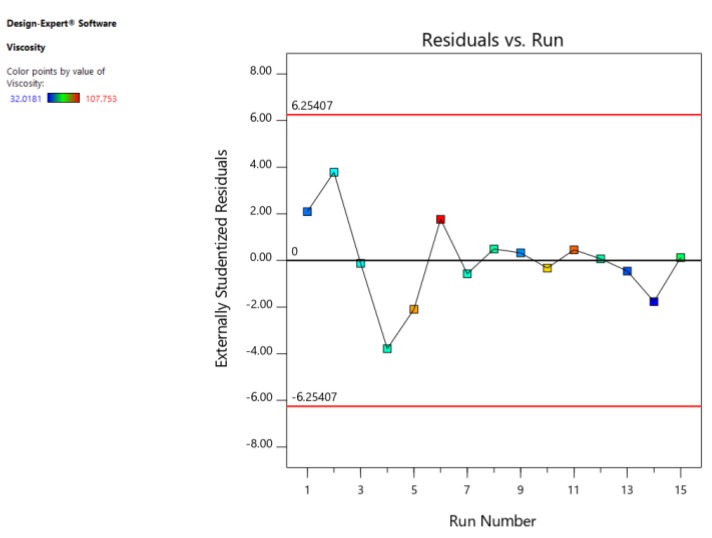
The plot of residuals versus the experimental run order.

The Box–Behnken experimental design was used in the design of experiments. Three selected influential factors, each set at three different levels, require 15 experiments to be randomly conducted. The corresponding response apparent viscosity of each prepared suspension was measured (Table 1). Then, the RSM was used to find the optimal value of each factor for minimum viscosity. The residual graphs for testing the model are shown in Appendix A. The first step was to examine the normal probability plot of the residuals. The normal probability plot of residuals versus predicted response values (Figure A2) indicates that the residuals follow a normal distribution. The plot of residuals versus the experimental run verifies the existence of a systematic error that could affect the response when conducting the experiments. Such a plot is shown in Figure A3. It is clear that there isn’t any systematic error present in the background measurements.

### 2.3. Preparation and Characterization of Al_2_O_3_—Waste Al_2_O_3_ Suspensions

Each suspension was prepared by first dissolving PVA in deionized water heated up to 80 °C. Then, while stirring, the dispersant and alumina powders were added. Suspension homogenization was performed in the planetary ball mill (PM 100, Retsch, Haan, Germany) for 90 min at 300 rpm. Grinding jar and balls were made of alumina in order to avoid any possible suspension contamination during ball milling. The prepared suspensions were treated with the ultrasound using the Branson B-220 Ultrasonic Cleaner (Branson, MO, USA) for the purpose of removing possible air bubbles and accomplishing more homogeneous suspensions. After the homogenization, the stability of water-based alumina suspensions was evaluated by the rheological testing. The characteristic rheological flow curve was recorded: apparent viscosity (*η*) versus shearing rate (*γ̇*).

One of the goals of this research was to determine the amount of each additive that will enable the minimal apparent viscosity at the shearing rate (*γ̇*) of 50 s^−1^, which is considered to be the shearing rate that occurs in the process of the common (gravity) slip casting.

DV-III Ultra Rheometer (AMETEK Brookfield, Middleboro, MA, USA) with the spindle SC4-18 was used for measuring the rheological properties. A pre-shear procedure was applied for 2 min at a 100 s^−1^ shearing rate. After the pre-shear, the suspensions were rested for 2 min in order to achieve repeatable results. The shearing rate was progressively increased from 0.1 to 500 s^−1^. The rheological testing was done before each shearing rate change. ECO RE 415 SW Cooling thermostat (LAUDA-Brinkmann, Delran, NJ, USA) was used in order to keep the temperature of the suspension stable at 25 ± 1 °C.

The stability of water-based alumina suspensions was also assessed by measuring the zeta potential of the suspensions, as well as by studying the sedimentation (settlement).

The zeta potential was measured using the zetasizer ZetaPALS (Brookhaven Instruments Corporation, Holtsville, NY, USA). The alumina suspension stability was investigated at different pH values. The samples for the zeta potential measurement were prepared with solid content of 0.02 vol.% by diluting the previously prepared 70 wt.% (≈40 vol.%) alumina suspension in a water based NaCl 0.001 M electrolytic solution. The pH modification was done by using water-based solutions of either hydrochloric acid (HCl) or sodium hydroxide (NaOH). A pH meter (Mettler Toledo, Toledo, OH, USA), calibrated by buffer solutions of pH 2, 4, 7 and 11 (Merck, Darmstadt, Germany) was used for measuring the pH value.

Sedimentation tests were carried out in a wide range of pH values (2, 3, 6, 8, 10 and 12) with the optimum dosage of additives in order to identify the effect of the pH value change on the stability of the 70 wt.% water-based alumina suspensions. After the homogenization by stirring at a rate of 1000 rpm for 45 min and the pH value adjustment, each suspension was transferred to 15 mL Eppendorf conical tubes, where they were left to stand undisturbed for one week. The volume of the sediment was recorded after 15 min, 1 h and after 1, 2, 3, 4 and 7 days.

## 3. Results and Discussion

The results of experiments were analyzed in order to investigate the influence of the commercial dispersant Tiron, PVA—poly (vinyl alcohol) as binder and magnesium aluminate spinel (MgAl_2_O_4_) as sintering additive on the stability of 70 wt.% alumina suspensions with a fixed weight ratio of waste alumina powder set to 20 wt.%. The preliminary results are important as they give an explanation for the weight ratio of waste alumina used in this research and the dispersant Tiron range used in the design of experiments. The optimal composition for the investigated factors was determined by the response surface methodology (RSM). The stability of the suspension with defined optimal composition was investigated by conducting the zeta potential measurement and the sedimentation tests. The microstructure of the samples with different addition of magnesium aluminate spinel was investigated in order to examine whether the abnormal grain growth of alumina will occur during the sintering process.

### 3.1. Preliminary Results

The use of the dispersant Tiron was preliminarily investigated for the stabilization of highly concentrated alumina suspensions with three different compositions of waste alumina powder: 15 wt.%, 20 wt.% and 25 wt.% expressed on a dry weight basis of total alumina powder. When adding an even higher amount of waste alumina, like 30 wt.%, it was not possible to prepare a stable homogenous suspension and this mixture was too viscous for the slip casting technique.

In order to define the region of interest, the dispersant amount was varied between 0.04 to 0.2 wt.% (Table A2). The rheological curves were recorded in order to determine the optimal dispersant amount, which accounts for the lowest achievable apparent viscosity at a shear rate of 50 s^−1^.

**Figure 1:**
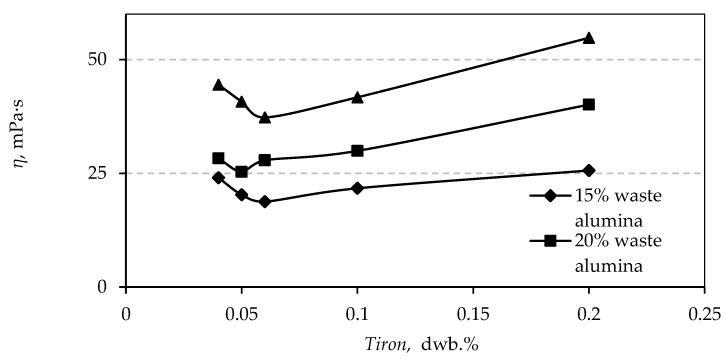
Apparent viscosity of alumina 70 wt.% suspensions with three different amounts of waste alumina powder as a function of Tiron concentration (dwb.%).

**Figure A4:**
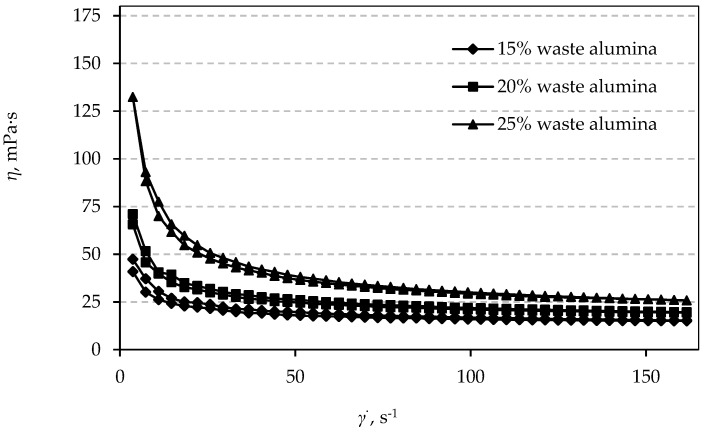
Variation of apparent viscosity of 70 wt.% suspensions with three different composition of waste alumina powder as a function of increasing and decreasing shear rate.

The addition of 20 wt.% of waste alumina powder expressed on a dry weight basis of total alumina powder mass has been chosen as a compromise between relatively low apparent viscosity and the amount of recycled waste alumina powder as shown in Figure 1 and Figure A4.

The results presented in Figure 1 indicate that the optimal amount of the dispersant Tiron suitable for slip casting is in the range between 0.05 and 0.06 wt.% for all three different compositions of prepared suspensions. Based on these preliminary results, the range of the dispersant Tiron was set from 0.05 to 0.15 wt.% for further research.

The upper and lower limits of the binder PVA—poly (vinyl alcohol) were set from 0.1 to 0.5 wt.% and for magnesium aluminate spinel (MgAl_2_O_4_) from 0.2 to 1 wt.%, all expressed on dry weight basis of the total alumina powder mass, by consulting the literature sources [22,24,25,26].

### 3.2. Results of Modeling

The corresponding response apparent viscosity of each prepared suspension was measured (Table 1). Then, the RSM was used to find the optimal value of each factor to obtain minimum apparent viscosity.

The predicted apparent viscosity (Table 1) was calculated by applying a second order polynomial equation to the experimental data. The following quadratic equation was obtained using the multiple regression analysis:

(1) $$\begin{array}{l} \mathrm{Apparent}\;\mathrm{viscosity}\;\left(\mathrm{mPa}\cdot s\right)\\ \;=24.91878+177.86637*A-59.1614*B+11.37873*C+142.834390*A*B\\ \;-112.01277*A*C+7.86044*B*C-236.73331*A^{2}+318.05723*B^{2}-1.93004*C^{2} \end{array}$$

where:

- *A* is the weight ratio of the dispersant Tiron (dwb.%),
- *B* is the weight ratio of the binder PVA (dwb.%),
- *C* is the weight ratio of the abnormal grain growth inhibitor MgAl_2_O_4_ (dwb.%).

The ANOVA data for the response surface quadratic model of the suspension apparent viscosity (Table 2) was analysed. A higher *F*-value (84.05) for the polynomial model and the related low *p*-value (i.e., *p* below 0.0001) confirmed that the selected regression model is adequate to assess the best amount of selected additives for the preparation of alumina suspensions, which contain 20 dwb.% waste alumina powder with moderately low apparent viscosity for easier mold filling. The *p*-value for the variable *B* (weight ratio of the binder PVA) was less than 0.0001 indicating that the change in the binder content had a significant effect on the apparent viscosity of the prepared alumina suspensions. The remaining two variables (the content of the dispersant Tiron and of the magnesium aluminate spinel, the grain growth inhibitor) had a relatively lower effect on the obtained apparent viscosity since their *p*-values were above 0.0001. High values of the coefficient of determination, *R*^2^ (0.9934), the adjusted *R*^2^ (0.9816) and the predicted *R*^2^ (0.9030) show that the predicted apparent viscosity fits adequately to the measured apparent viscosity by using the selected fitting model. The predicted *R*^2^ (0.9030) is also quite high, which shows that the proposed regression model may adequately predict responses for new experimental runs. The coefficient of variation *C.V.* being below 10% (5.24%) shows that the presented model offers a high precision too. It also indicates the reliability of the performed experimental runs.

After setting up the minimum apparent viscosity as the optimization criterion, with three independent variables set in previously defined data ranges, the best solution to the apparent viscosity was at 0.05% Tiron, 0.1% PVA and 0.2% MgAl_2_O_4_ dwb. with a desirability of 0.994.

### 3.3. Effect of Additives on Stability of Alumina—Waste Alumina Suspension

**Figure 2:**
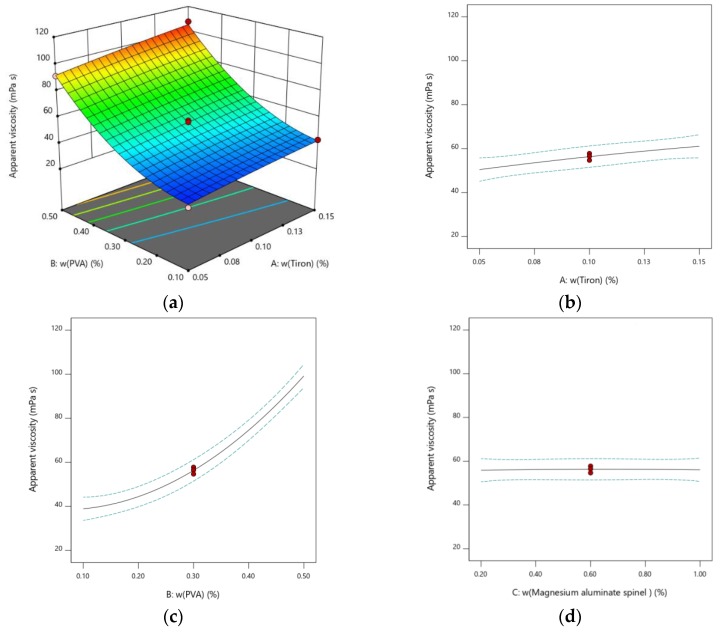
The effect on apparent viscosity of 70 wt.% Al_2_O_3_ suspensions which contain 20 dwb.% waste Al_2_O_3_ powder: (**a**) 3D-plot of viscosity on two factors (PVA content and Tiron content); (**b**) Tiron; (**c**) poly (vinyl alcohol) (PVA); (**d**) magnesium aluminate spinel (MgAl_2_O_4_).

Studies on dispersing the alumina particles using low molecular weight organic dispersants such as Tiron for the preparation of highly concentrated stable alumina suspensions have been reported [18,19]. It is crucial to determine the most adequate amount of the dispersant for achieving minimum apparent viscosity of stable alumina suspensions. Any percentage higher or lower than the optimum amount increases the apparent viscosity, which in turn results in the suspension’s instability. An increase in Tiron content for the investigated range has shown a slight impact on the increase in the apparent viscosity. The *p*-value of this variable was 0.0060 (Table 2 and Figure 2b).

Poly (vinyl alcohol) (PVA) is a hydro soluble organic binder. PVA, when added to the ceramic suspensions, enhances the strength of the ceramic green body [22]. An increase in the amount of added PVA increases the apparent viscosity of the suspension. Even though an increase in the amount of PVA has a positive influence on the strength of the green body, the increase in content of PVA negatively affects the stability of the prepared suspensions by increasing the apparent viscosity of the suspension. The calculated *p*-value for the PVA variable was less than 0.0001, indicating that the change in the content of poly (vinyl alcohol) had an important effect on the apparent viscosity of the prepared alumina suspensions (Table 2 and Figure 2c).

Magnesium aluminate spinel (MgAl_2_O_4_) is usually added to inhibit the abnormal alumina grain growth during the sintering process of the green bodies [27]. The increase in the content of magnesium spinel has not shown any effect on the apparent viscosity of the suspension in the investigated range. This statement was confirmed by the *p*-value which was above 0.0001 (Table 2 and Figure 2d).

**Figure 3:**
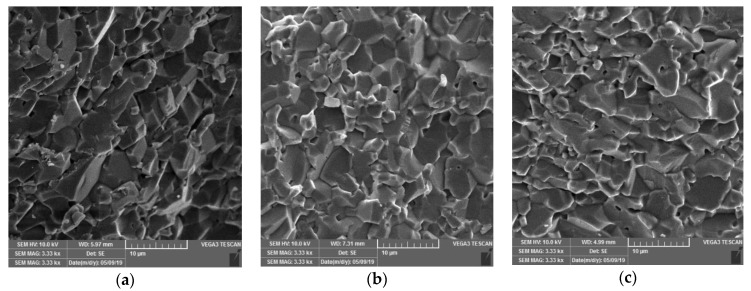
SEM images of alumina morphology sintered at 1550 °C with fixed weight ratios of waste alumina powder of 20 wt.%, 0.05 wt.% Tiron and 0.1 wt.% PVA and with varying amounts of magnesium aluminate spinel: (**a**) 0.2 wt.% MgAl_2_O_4_, (**b**) 0.6 wt.% MgAl_2_O_4_ and (**c**) 1.0 wt.% MgAl_2_O_4_ expressed on dry weight basis.

**Figure A5:**
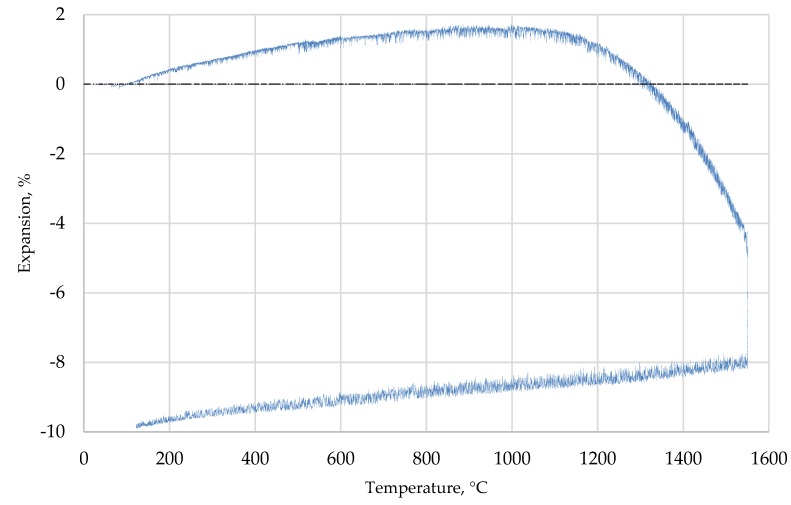
The effect of sintering temperature on the densification of the sintered sample.

Finally, the prepared suspensions were cast in gypsum molds (21 mm × 21 mm × 21 mm) to prepare the green bodies. The sintering shrinkage behavior for the optimal suspension composition was investigated by using an optical dilatometer (MISURA, Expert System Solutions, Comune di Modena, Italy). The effect of the sintering regime on the densification of the sintered sample is shown in Figure A5. The green bodies were conventionally sintered (CS) in an electric resistance heating furnace at 1550 °C. The heating rate of 5 °C per minute and the holding time of 2 h were programmed. The density of the sintered samples was determined in distilled water by the Archimedean method. The theoretical density of alumina ceramics, used for the calculation of the relative density, was taken as 3.987 g·cm^−3^. The microstructure of the samples with different additions of magnesium aluminate spinel was investigated by scanning electron microscopy (Tescan Vega3, Brno, Czech Republic) in order to examine whether the abnormal alumina grain growth will occur during the sintering process (Figure 3).

Scanning electron microscopy (SEM) was used to study the fracture surface of alumina—waste alumina samples. The microstructure of the examined samples revealed a satisfactory bonding between the alumina particles. Also, the samples showed a similar morphology with grains of irregular shape, sharp edges and random orientation. For all examined samples, the grain size was in the range between 1–10 µm. The relative density for all three samples was 97.2 ± 0.4%. It can be concluded from the SEM images that the abnormal grain growth has not occurred. The addition of 0.2 wt.% MgAl_2_O_4_ was confirmed as optimal from the economic point of view. The relative density of the obtained sample with optimal composition was calculated to be 97.1%.

### 3.4. Rheological Properties

**Figure 4:**
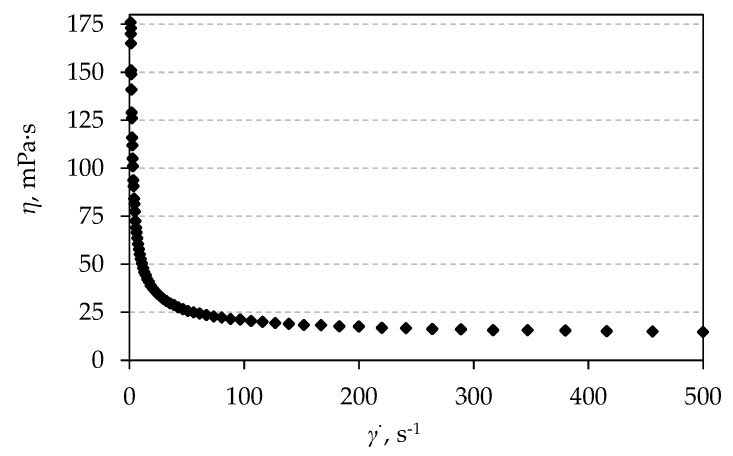
Plot of apparent viscosity (*η*) versus increasing shearing rate (*γ̇*) for 70 wt.% Al_2_O_3_ water based suspension which contains 20 dwb.% of waste Al_2_O_3_ powder, with optimal amounts of additives.

The stability of the ceramic suspension should be met for a good control over its rheological properties. The suspension stability is frequently evaluated through viscosity measurements. The stability of the water-based suspension with optimal amounts of additives was assessed by tracking the apparent viscosity at various shearing rates, ranging from 1–500 s^−1^. The rheological behavior of the prepared ceramic suspensions estimated by the apparent viscosity versus shearing rate is shown in Figure 4.

The obtained results showed that the apparent viscosity of the suspension decreases while the shearing rate increases (Figure 4). This is a characteristic of the non-Newtonian fluids, or more precisely, the pseudoplastic behavior of fluids [28].

### 3.5. Zeta Potential Measurements

**Figure 5:**
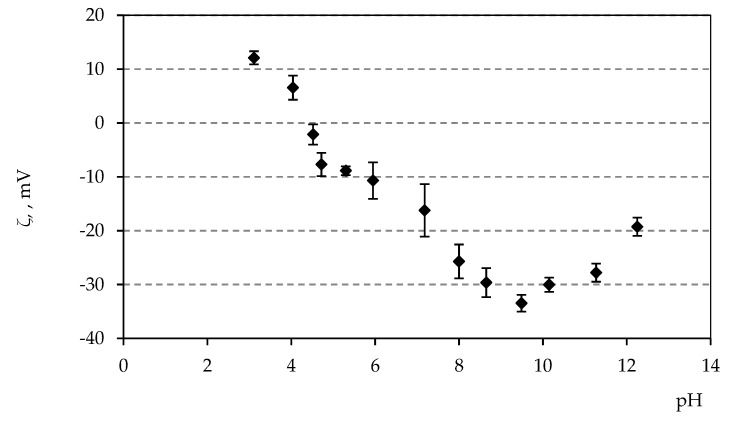
Zeta potential vs. pH values of alumina suspension which contains optimal amounts of additives.

The electrokinetic potential or the so-called zeta potential is built on alumina particles when they come in contact with water. The addition of additives (0.05 dwb.% Tiron, 0.1 dwb.% PVA and 0.2 dwb.% magnesium aluminate spinel) to the concentrated alumina suspensions changes the alumina surface charge properties. Strong repellant forces are present between the alumina particles as a result of the highly negative surface charge generated by the adsorption and dissociation of Tiron molecules in water-based alumina suspensions. The stability of concentrated suspensions is generally assumed when the absolute value of the zeta potential is above 30 mV [29]. The zeta potential of alumina suspensions which contain the optimal amount of additives was measured using electrophoresis at various pH values, as shown in Figure 5, with the pH values adjusted by HCl or NaOH.

The isoelectric point (IEP) of the zeta potential means that the potential is zero, i.e., there isn’t any electric charge present on the particle surface. The IEP was determined at a pH value around 4.5 for the investigated optimal composition. The stability of suspensions, i.e., the zeta potential value |ζ| > 30 mV, was achieved when the pH value of the suspension was between 8–11. The magnitude of the negative zeta potential value was higher and extended in the alkaline pH range, which can be attributed to impurities from waste alumina powder [30].

### 3.6. Sedimentation Tests

**Figure 6:**
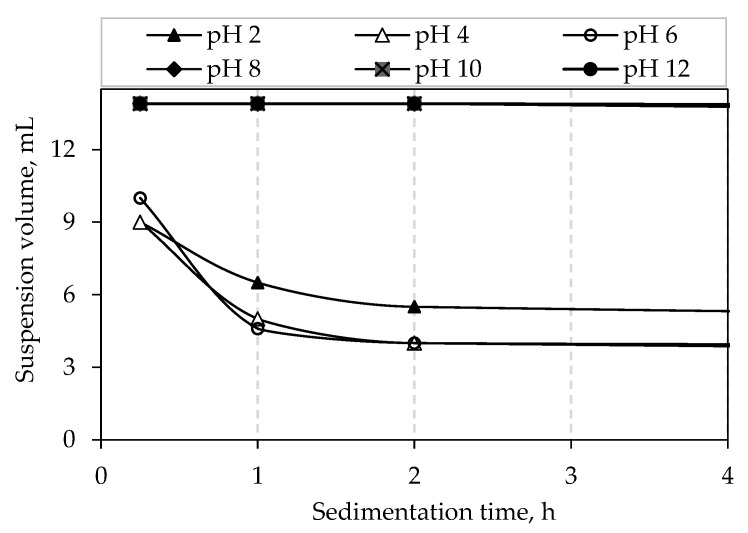
Sedimentation rate of water-based alumina suspensions with optimal composition in the first hours after preparation.

The sedimentation test was carried out in order to validate the electrophoretic measurements, i.e., the zeta potential measurements. The dispersion height measured during the sedimentation test decreases rapidly if the optimal pH value of the suspension is not reached. As it was shown in Figure 5, the alumina suspension with the optimal amount of additives was stable in the pH range from 8–11. After the optimal pH value is achieved, the dispersion height typically remains constant for a certain period of time and then slowly decreases [31]. The sedimentation tests, based on visual observation of the suspension sedimentation consisted of recording the sediment volume after 15 min and then after 1 and 2 h and after 1, 2, 3, 4 and 7 days. The suspensions with the optimal amount of additives, but which were in the pH range below 8, have started to sediment rapidly (Figure 6). On the other hand, the suspensions that had the pH value of 8, 10 or 12 have not showed almost any sedimentation in the first two hours after the preparation. Some plot markers overlap in the upper segment of Figure 6.

**Figure 7:**
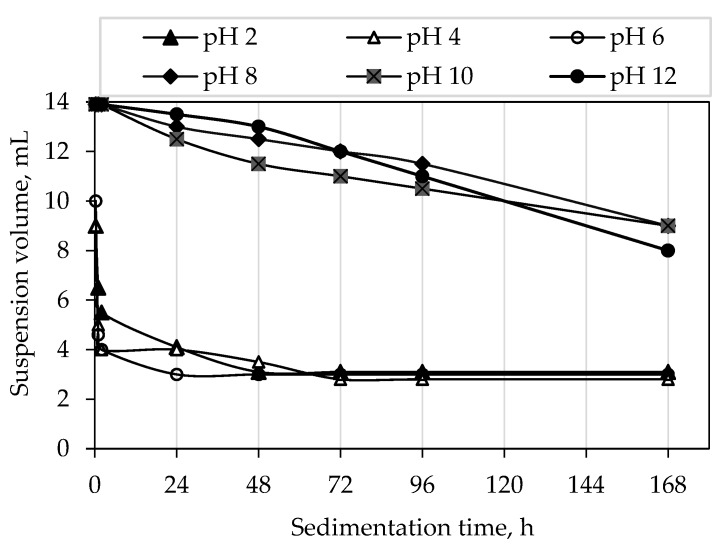
Sedimentation rate of water-based alumina suspensions with optimal composition after 168 h (7 days).

After 72 h the sedimentation process was completed for the suspensions with the pH values below 8, which suggests highly unstable suspensions. The suspensions which were in the pH range above 8 showed a reasonable stability after 72 h. For these suspensions a complete sedimentation was not observed even 7 days (168 h) after the preparation (Figure 7).

## 4. Conclusions

The possibility of recycling waste alumina powder, which is obtained by green machining in the factory production of technical ceramics, was investigated. Three independent variables were considered for the preparation of stable highly-concentrated (70 wt.%) alumina suspensions which contain 20 dwb.% of waste alumina powder. The Box-Behnken response surface methodology was used to optimize the amounts of additives in the water based ceramic suspensions. The minimum apparent viscosity was determined by numerical optimization using Design Expert software. The optimum parameters for a minimum apparent viscosity (32.43 mPa·s) of the investigated suspensions were achieved with the addition of 0.05 wt.% Tiron, 0.1 wt.% PVA and 0.2 wt.% magnesium aluminate spinel (MgAl_2_O_4_), as per the dry weight bases of total alumina powders. The polynomial regression model suggests that adding PVA to the suspensions has an important effect on apparent viscosity, whereas the addition of Tiron and MgAl_2_O_4_ suggested a relatively lesser impact on the apparent viscosity of the prepared alumina suspensions. The apparent viscosity was also used for the evaluation of the suspension stability. The stability of the suspension with the defined optimal composition was additionally investigated by conducting the zeta potential measurements and the sedimentations tests. Both testing results indicate that the prepared suspensions are stable in the pH range from 8–11.

This study indicates that it might be possible to recycle waste alumina powder for the industrial production of ceramics, as stabilized suspensions with reasonably low apparent viscosity were prepared. The study was limited to the preparation of stable alumina suspensions adequate for the slip casting ceramic forming technique with a fixed ratio of waste alumina powder of 20 dwb.% of total alumina powder mass.

The next step in this research would be to measure the mechanical properties, like hardness and fracture toughness, after sintering the green bodies. These properties would be the most important to assess if novel ceramic products with added waste alumina could be made and used. The proposed recycling of waste alumina powder would help in saving the manufacturing and waste disposal costs, and also in reducing the landfilled amounts.

## Figures and Tables

**Table 1:** Experimental results.

Experiment	Factor *A*: Tiron, dwb.%	Factor *B*: PVA dwb.%	Factor *C*: MgAl_2_O_4_ dwb.%	Response Apparent Viscosity mPa·s	Predicted Apparent Viscosity mPa·s
1	0.1	0.1	1	40.72	38.06
2	0.05	0.3	0.2	51.03	47.79
3	0.05	0.3	1	52.22	52.45
4	0.15	0.3	1	55.34	58.58
5	0.1	0.5	0.2	95.51	98.16
6	0.15	0.5	0.6	107.75	105.33
7	0.1	0.3	0.6	54.68	56.33
8	0.1	0.3	0.6	57.76	56.33
9	0.15	0.1	0.6	42.79	42.20
10	0.05	0.5	0.6	91.26	91.86
11	0.1	0.5	1	100.41	99.59
12	0.1	0.3	0.6	56.53	56.33
13	0.1	0.1	0.2	38.33	39.15
14	0.05	0.1	0.6	32.02	34.44
15	0.15	0.3	0.2	63.11	62.89

**Table 2:** ANOVA for response surface quadratic model for apparent viscosity of suspension.

Source	Sum of Squares	df *	Mean Square	*F*-Value	*p*-Value
Model	8132.05	9	903.56	84.05	< 0.0001
A (Tiron)	225.42	1	225.42	20.97	0.0060
B (PVA)	7264.81	1	7264.81	675.76	< 0.0001
C (MgAl_2_O_4_)	0.062	1	0.062	0.005738	0.9426
AB	8.16	1	8.16	0.76	0.4234
AC	20.07	1	20.07	1.87	0.2300
BC	1.58	1	1.58	0.15	0.7171
A^2^	1.29	1	1.29	0.12	0.7428
B^2^	597.62	1	597.62	55.59	0.0007
C^2^	0.35	1	0.35	0.033	0.8635
Residual	53.75	5	10.75		
Pure Error	4.80	2	2.40		
Cor. Total	8185.80	14		*R* ^2^	0.9934
Std. Dev.	3.28			Adjusted *R*^2^	0.9816
Mean	62.63			Predicted *R*^2^	0.9030
C.V. %	5.24			Adequate Precision	26.478

* df—degree of freedom.

## Appendix A

**Table A1:** Composition of prepared suspensions used in preliminary experiments.

Dispersant	High-Purity Al_2_O_3_ Dry Powder Content (wt.%)	Waste Al_2_O_3_ Dry Powder Content (wt.%)	Water Content (wt.%)	* Dispersant Content (dwb.%)
Tiron	55	15	30	0.04–0.1
	50	20	30	0.04–0.1
	45	25	30	0.04–0.1

* Expressed on dry weight basis (dwb.) of alumina powder.

**Table A2:** Measured apparent viscosity of all prepared suspensions.

Waste Al_2_O_3_ Dry Powder Content dwb.%	Tiron dwb.%	pH	η (mPa·s)	
			γ = 50 s^−1^	γ = 100 s^−1^
**15**	**0.04**	8.78	22.63	20.04
	0.05	8.79	19.32	17.20
	0.06	8.75	17.93	15.97
	0.1	8.64	20.40	17.76
	0.2	8.41	25.62	21.19
20	0.04	8.86	27.09	23.55
	0.05	8.89	24.63	21.11
	0.06	8.86	26.71	22.83
	0.1	8.72	29.17	23.99
	0.2	8.57	40.10	29.18
25	0.04	8.96	43.33	35.16
	0.05	8.98	39.71	32.21
	0.06	8.92	36.48	29.34
	0.1	8.80	43.10	34.01
	0.2	8.43	54.79	39.21
